# Hemoglobin Video Imaging Provides Novel In Vivo High-Resolution Imaging and Quantification of Human Aqueous Outflow in Patients with Glaucoma

**DOI:** 10.1016/j.ogla.2019.04.001

**Published:** 2019

**Authors:** Tasneem Z. Khatib, Paul A.R. Meyer, Jed Lusthaus, Ilya Manyakin, Yusuf Mushtaq, Keith R. Martin

**Affiliations:** 1 John van Geest Centre for Brain Repair, University of Cambridge, Cambridge, United Kingdom; 2 Eye Department, Cambridge University Hospitals NHS Foundation Trust, Cambridge, United Kingdom; 3 Sydney Eye Hospital Glaucoma Unit, Sydney, Australia; 4 Discipline of Ophthalmology, The University of Sydney, Sydney, Australia; 5 Department of Physics, University of Cambridge, Cambridge, United Kingdom; 6 School of Clinical Medicine, University of Cambridge, Cambridge, United Kingdom; 7 Cambridge NIHR Biomedical Research Centre, Cambridge, United Kingdom; 8 Wellcome Trust – MRC Cambridge Stem Cell Institute, University of Cambridge, Cambridge, United Kingdom

**Keywords:** CSA, cross-sectional area, HVI, hemoglobin video imaging, IOP, intraocular pressure, MD, mean deviation, MIGS, minimally invasive glaucoma surgery, SLT, selective laser trabeculoplasty

## Abstract

**Purpose:**

Noninvasive, detailed measurement of the dynamics of human aqueous outflow is difficult to achieve with currently available clinical tools. We used hemoglobin video imaging (HVI) to develop a technique to image and quantify human aqueous outflow noninvasively and in real time.

**Design:**

A prospective observational study to describe characteristics of aqueous veins and a pilot prospective interventional feasibility study to develop quantification parameters.

**Participants:**

Patients were recruited from the Cambridge University Hospitals NHS Foundation Trust Glaucoma clinic. The observational study included 30 eyes, and the pilot interventional feasibility study was performed on 8 eyes undergoing selective laser trabeculoplasty (SLT). Our SLT protocol also included the installation of pilocarpine and apraclonidine eye drops.

**Methods:**

Participants underwent HVI alongside their usual clinic visit.

**Main Outcome Measures:**

The change in cross-sectional area (CSA) of the aqueous column within episcleral veins was correlated with intraocular pressure (IOP) reduction and change in visual field mean deviation (MD) before and after intervention. Fluctuations in contrast and pixel intensity of red blood cells in an aqueous vein were calculated to compare the flow rate before and after intervention using autocorrelation analysis.

**Results:**

Hemoglobin video imaging enables the direct observation of aqueous flow into the vascular system. Aqueous is seen to centralize within a laminar venous column. Flow is pulsatile, and fluctuations of flow through globe pressure or compression of the aqueous vein are observed. There was a significant increase in the aqueous column after the administration of our SLT protocol (n = 13; *P* < 0.05). This correlated with the degree of IOP reduction (n = 13; Pearson’s correlation coefficient 0.7; *P* = 0.007) and the improvement in MD observed postintervention (n = 8; Pearson’s correlation coefficient 0.75; *P* = 0.03). Autocorrelation analysis demonstrated a faster rate of decay in an aqueous vein after intervention, indicating an increase in flow rate.

**Conclusions:**

Hemoglobin video imaging can be incorporated into a routine clinic slit-lamp examination to allow a detailed assessment and quantification of aqueous outflow in real time. It has the potential to be used to help target therapeutic interventions to improve aqueous outflow and further advance our understanding of aqueous outflow dysregulation in the pathogenesis of glaucoma.

Noninvasive, detailed measurement of the dynamics of human aqueous outflow is difficult to achieve with currently available clinical tools. Our knowledge of the anatomy and physiology of aqueous outflow is based on studies in ex vivo tissue^1^, ^2^, ^3^, ^4^ and in vivo techniques that are static^5^ or invasive or involve a degree of manipulation of physiologic parameters.^4^ The widely used fluorescein disappearance test^6^, ^7^ is at best an indirect estimate of aqueous outflow, and the outflow pathway cannot be visualized using this technique.

The advent of minimally invasive glaucoma surgery (MIGS) procedures has led to renewed interest in the dynamics of aqueous outflow. The intraocular pressure (IOP) lowering effect of trabecular bypass devices is variable in different patients.^8^, ^9^, ^10^, ^11^, ^12^ The segmental and dynamic nature of aqueous outflow have been described,^3^, ^4^ and it has been suggested that targeting trabecular bypass stents to ocular quadrants with good aqueous outflow could improve the success rates of these procedures.

We describe a technique to visualize aqueous veins noninvasively using hemoglobin video imaging (HVI),^13^ which uses the hemoglobin absorption spectrum to enhance the contrast between red blood cells and their surroundings. Erythrocytes are displayed as darker objects against a brighter background of light reflected by sclera with a resolution down to the level of a single red blood cell. Aqueous is observed as an erythrocyte void, a clear column that displaces red blood cells as it flows into the episcleral venous circulation.

We describe characteristics of aqueous veins that are consistent with earlier reports, and we have developed a quantification technique to measure the cross-sectional area (CSA) of the aqueous column within episcleral veins. As an example of the type of clinical investigation possible with our technique, we performed a pilot study on 8 eyes of 7 patients who underwent selective laser trabeculoplasty (SLT) together with administration of pilocarpine and apraclonidine eye drops, as per our standard protocol, and correlated the change in CSA with IOP reduction and change in visual field mean deviation (MD) before and after intervention. We also propose a method that could be used to compare the flow rate from HVI images before and after intervention.

Hemoglobin video imaging can be incorporated into a routine clinic slit-lamp examination to allow a detailed assessment of physiologic and pathologic aqueous outflow in real time. We suggest that HVI has the potential to be used as a tool to help target therapeutic interventions, improve aqueous outflow, and further advance our understanding of aqueous outflow dysregulation in the pathogenesis of glaucoma.

## Methods

The study was conducted in accordance with the tenets of the Declaration of Helsinki. The Institutional Review Board of Cambridge University Hospitals NHS Foundation Trust and the Local Research Ethics Committee approved the study (REC reference number: 15/LO/2171). All subjects gave written informed consent before participation in the study.

### Imaging Aqueous Veins in Human Eyes

We performed an observational study on 30 glaucomatous eyes to determine characteristics of aqueous veins using HVI. Images were captured using a monochromatic Prosilica GC1380H camera attached to a Zeiss SL130 slit lamp (Oberkochen, Germany). As described previously,^13^ the slit-lamp illumination system is fitted with a band-pass interference filter (steep long and short wavelength cutoff; >50% transmission between 505 and 575 nm) and a hot mirror that stops light with wavelengths beyond 730 nm from reaching the camera. The video camera is mounted on a 50% beam-splitter with a 220-mm focal length C-mount. Images are captured at 30 frames per second, without compression. During the live recording of aqueous veins, images are displayed in real time using bespoke HVI software.

### Image Processing

Raw image data were exported from the HVI software in .pgm format and processed using Image J. Image sequences were stabilized using the “Image J Stabilizer” plugin (Videos 1 and 2, available at www.ophthalmologyglaucoma.org) before quantification. We developed a computer model demonstrating the changes in transmitted light along an orthogonal transept of a vein containing a central aqueous column. The diameter of the column was found to be the distance (δ) between intensity minima. For calculation of the CSA, we assumed the vessel had a circular section. Measurements were made upstream of a vessel confluence. We assessed the repeatability of measurements by comparing values from 4 to 10 separate images per eye for 9 individuals. For extended-length sequences to enable detailed observation of aqueous vein characteristics, stabilization was performed using Adobe After Effects CC (version 15.1.12; San Jose, CA).

### Aqueous Column Cross-sectional Area Following Our Selective Laser Trabeculoplasty Protocol

We performed HVI on 8 eyes immediately before and 10 minutes after SLT. The SLT energy settings were 0.3 to 1.2 mJ targeting 90^o^ to 360^o^ of the trabecular meshwork. The IOP was measured immediately before and 30 minutes postprocedure. Pilocarpine nitrate 2% (Bausch & Lomb, Rochester, NY) and apraclonidine 1% (Alcon, Fort Worth, TX) eye drops were instilled 30 minutes before SLT. The CSA of the aqueous column was calculated and correlated with the degree of IOP reduction observed and the change in MD using 24-2 Humphrey visual field Swedish Interactive Threshold Algorithm standard testing pre- and postintervention. The mean length of time for MD measurement postintervention was 23 weeks (range, 15–30 weeks).

### Quantifying Flow Rate

Aqueous flows as a central stream through a column of venous blood. Therefore, enhancement of aqueous drainage can be expected to increase the flow rate of the surrounding red blood cells, and it is this effect that we aimed to exploit.

We used principles derived from photon correlation spectroscopy and laser Doppler velocimetry where fluctuations in the recorded intensity signal are examined to estimate parameters such as particle size or velocity. For aqueous flow using successive HVI images, fluctuations in contrast and pixel intensity of red blood cells through an aqueous vein were calculated and used to compare the rate of flow before and after our SLT protocol.

#### Quantifying Flow Rate: Fluctuation Transformation

The sum of the absolute values of pixel-wise differences from successive stabilized HVI frames were taken:

$$d_{ij}=\overset{N-1}{\underset{t=0}{\sum }}\left|p_{ij}\left(t+1\right)-p_{ij}\left(t\right)\right|$$

where *t* denotes frame number, (i, j) denotes pixel coordinates of the individual pixels and $p_{ij}\left(t\right)$ is the pixel intensity in frame t, with N being the total number of recorded frames and $d_{ij}$ being the un-normalized fluctuation value. To extract the relative scale of the fluctuations in comparison with the whole image, we then normalize the computed values as follows:

$$n_{ij}=\left(d_{ij}-\mu \right)/\sigma$$

where μ,σ are, respectively, the mean and standard deviations of the fluctuations values $d_{ij}$ in the image. The results of this transformation before and after the intervention are illustrated in Figure S1 (available at www.ophthalmologyglaucoma.org).

#### Quantifying Flow Rate: Autocorrelation Analysis

This transformation was used to segment the pixels of interest from the background and compute an “autocorrelation” to quantify the timescale of pixel fluctuations and how fast the pixel values change. Pixels with normalized fluctuation value above a given threshold of 2.5 were selected and the mask applied to all frames in the video.

$$R_{ij}\left(n\right)=\frac{E\left[\left(p_{ij}\left(t\right)-\mu )(p_{ij}\left(t+n\right)-\mu \right)\right]}{\sigma^{2}}$$

Where $R_{ij}\left(n\right)$ denotes the autocorrelation function value for a pixel at position (i, j) at a frame delay value *t*, μ is the mean pixel value in the segmented image, and σ is the standard deviation of the pixel values in the segment. The mean signal was computed by averaging the autocorrelation across all pixels. To show invariance, we also computed the autocorrelations for the background of the HVI images.

## Results

**Figure 1:**
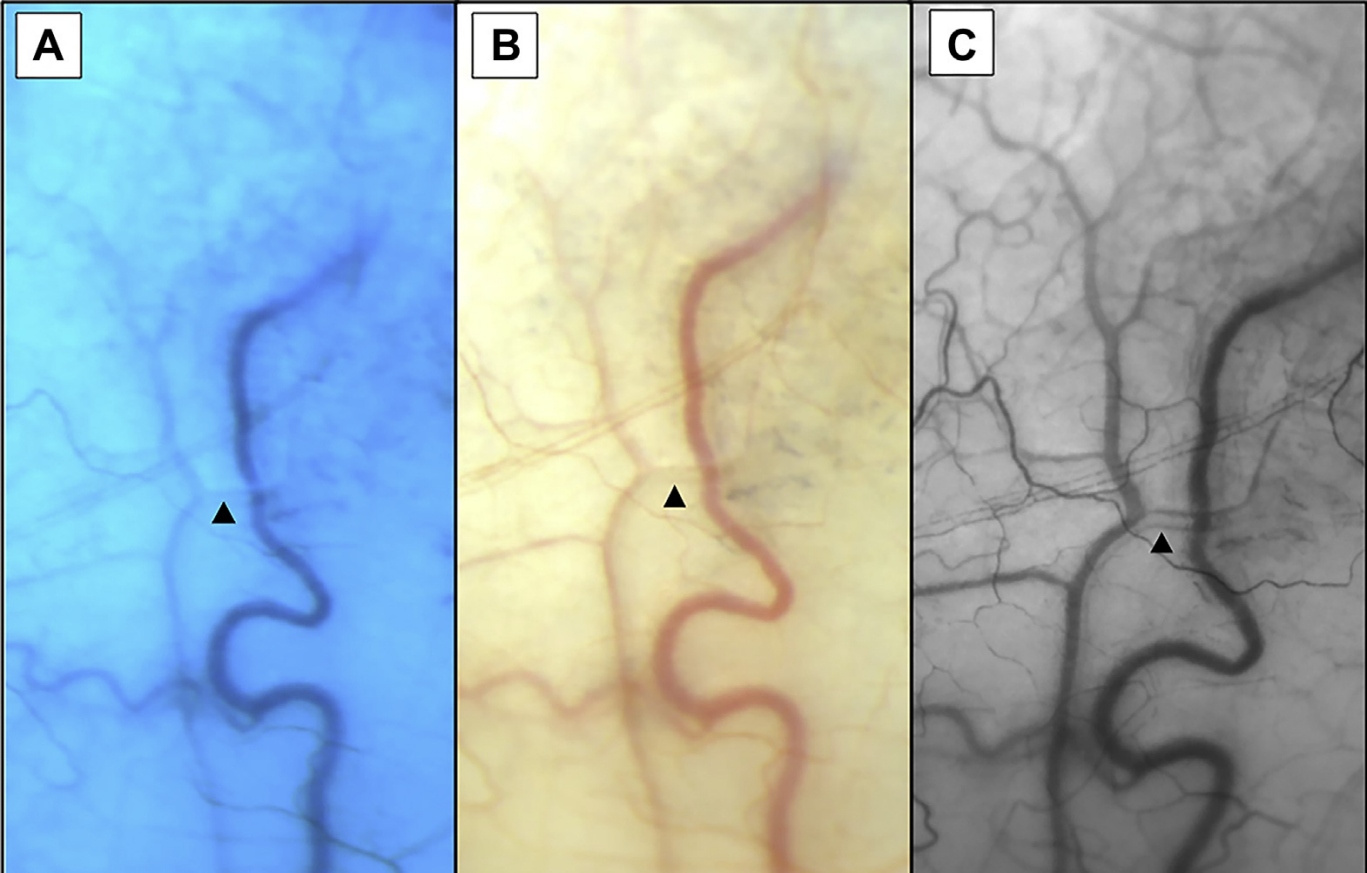
Aqueous vein (arrow) captured using conventional techniques (**A** and **B**) and hemoglobin video imaging (HVI) **(C)**.

The HVI technique demonstrates aqueous as an erythrocyte void at high contrast to hemoglobin in episcleral venous blood (Fig 1).

**Figure 2:**
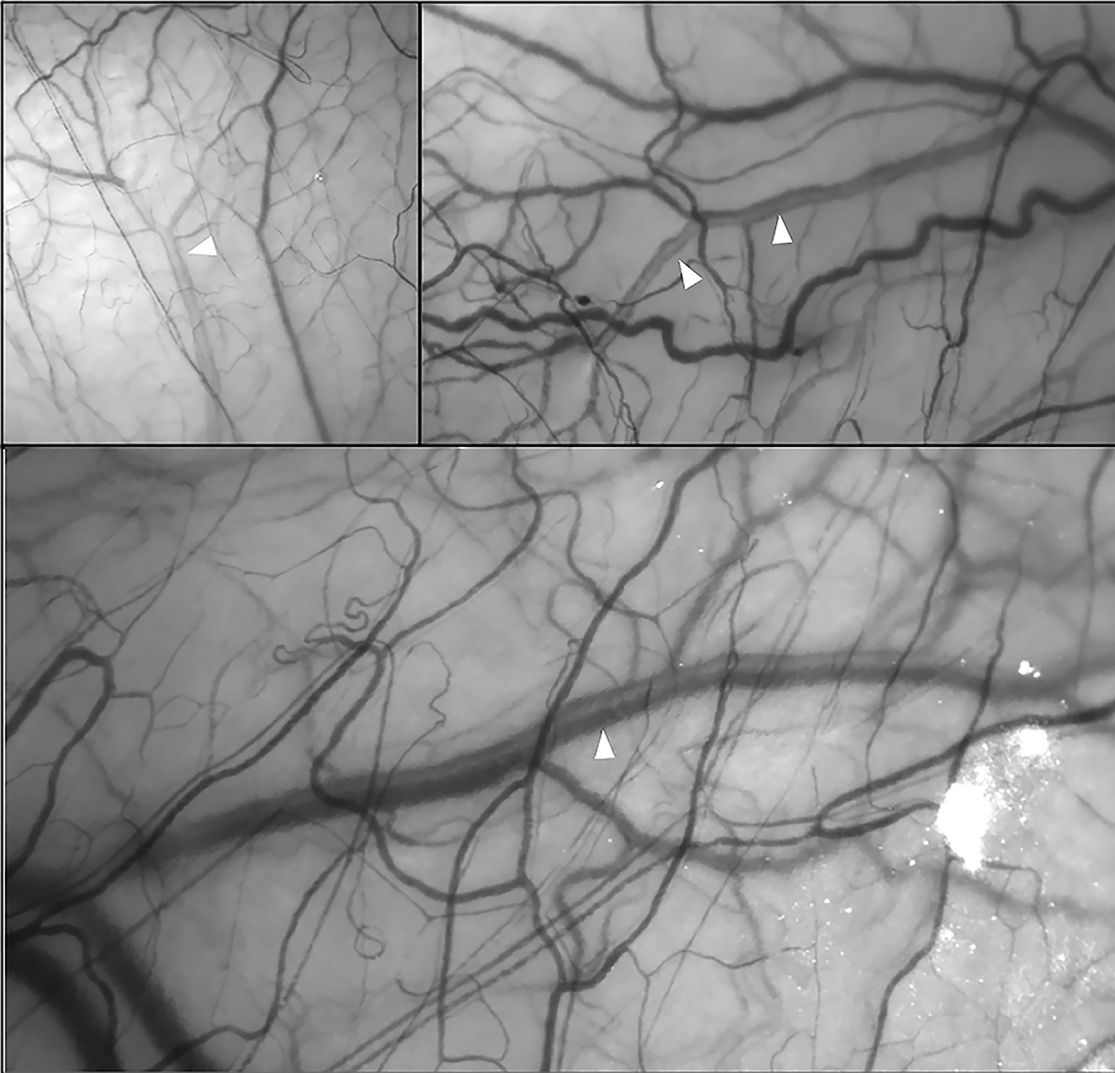
Examples of aqueous veins obtained using hemoglobin video imaging (HVI) (white arrows). Aqueous is seen as a centralized erythrocyte void.

In every vein observed, aqueous centralized within a laminar venous column, regardless of its point of entry into the episcleral circulation (Fig 2; Video 3, available at www.ophthalmologyglaucoma.org). A “*” symbol briefly appears to denote the vessel(s) of interest in each video within the clip before disappearing to permit uninterrupted observation of flow dynamics. A corresponding header also identifies the main observation to be made from individual recordings.

**Figure 3:**
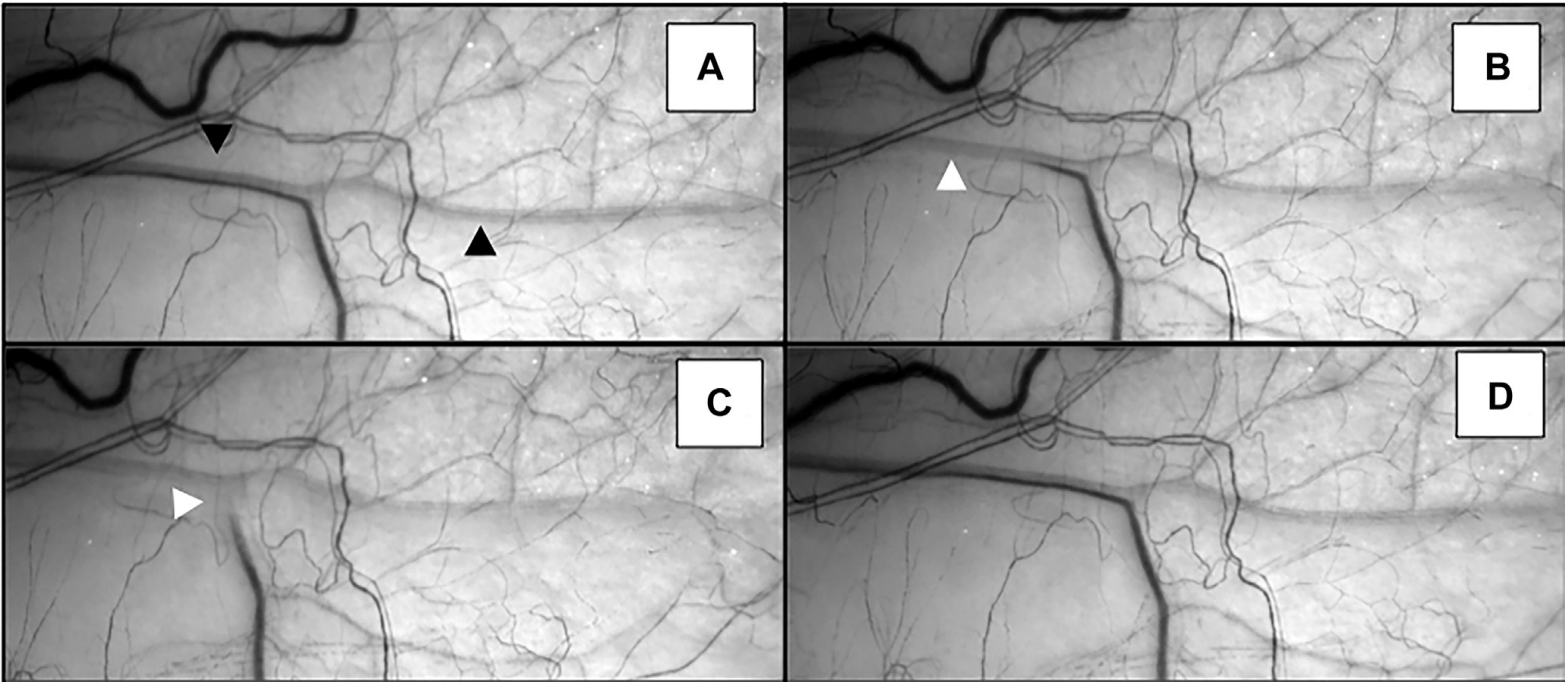
Displacement of aqueous after digital pressure on the inferior globe. **A,** Aqueous vein (black arrow) before digital manipulation. **B** and **C,** Aqueous is redirected into an episcleral blood filled vessel after digital pressure on the globe (white arrow). **D,** Immediate resumption of usual aqueous and blood flow after release of pressure.

**Figure 4:**
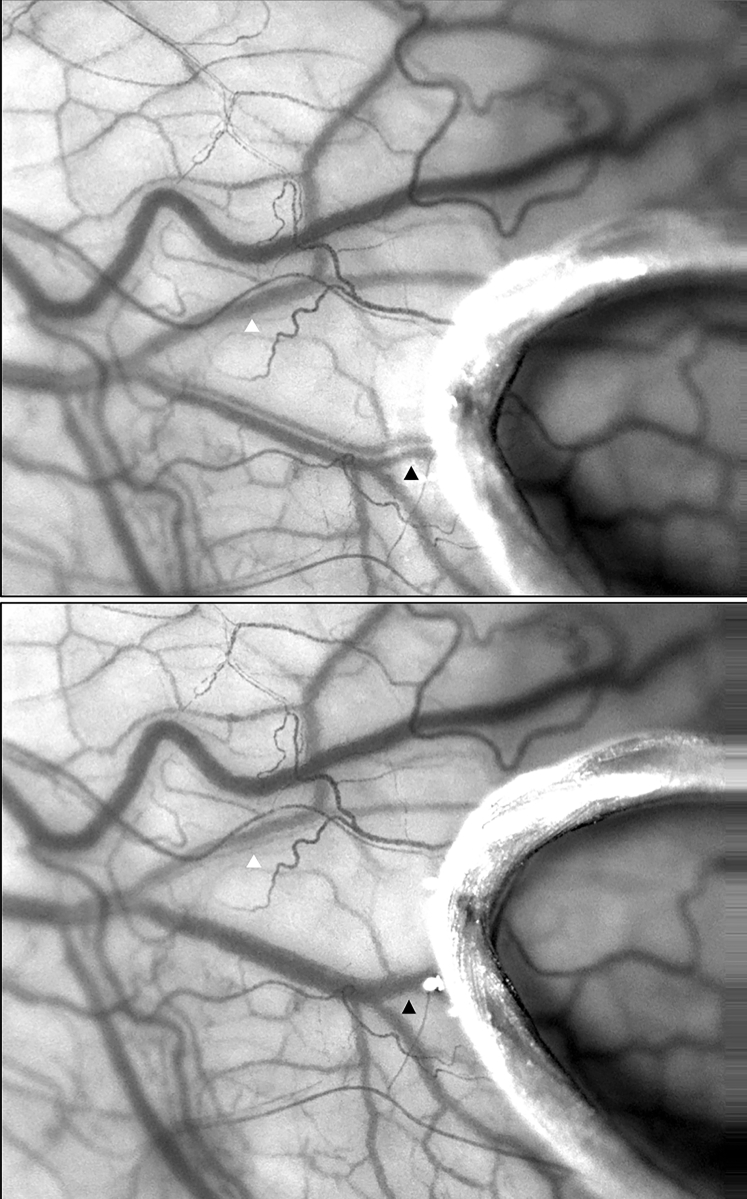
Compression of an aqueous vein (white arrow) using a 10/0 Vicryl loop redirects aqueous to a nearby episcleral blood vessel (black arrow).

The length and diameter of aqueous streams varied, but some continued beyond the conjunctival reflection. Fluctuations arose in the aqueous stream, corresponding with cardiac rhythm, eye movements, and pressure on the globe (Fig 3; Video 4, available at www.ophthalmologyglaucoma.org). Compression of the aqueous vein resulted in the redirection of aqueous flow to other vessels that had previously been filled with blood (Fig 4; Video 4, available at www.ophthalmologyglaucoma.org).

**Figure 5:**
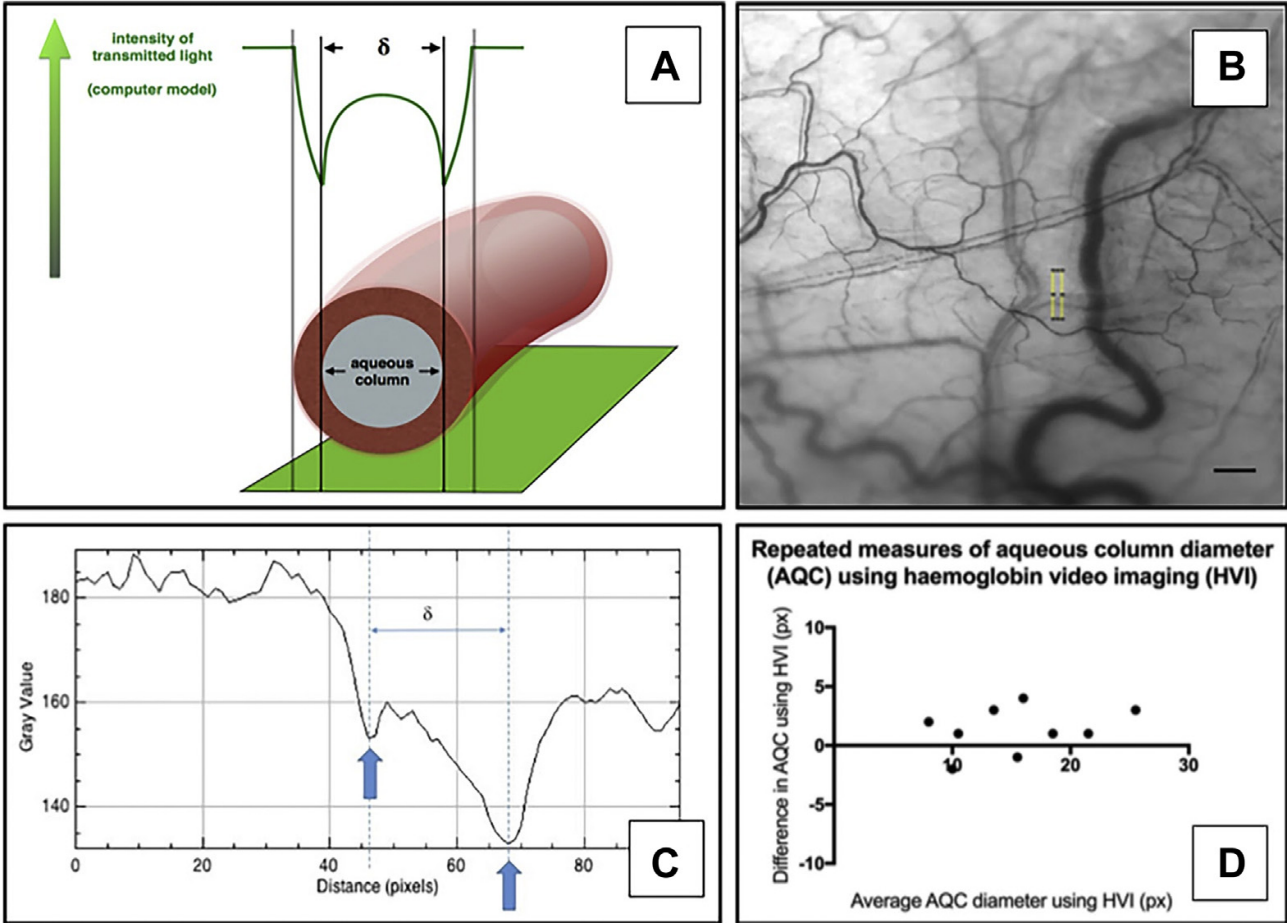
**A,** Schematic representation of the intensity profiles of transmitted light in an aqueous vein using hemoglobin video imaging (HVI). **B** and **C,** Aqueous vein transept with corresponding density profile and δ measurement. Scale bar = 0.5 mm. **D,** Bland–Altman plot of the difference in paired δ measurements using HVI against the mean δ measurement.

The CSA calculations arising from δ (Fig 5) were consistent and repeatable for each eye measured. Any variation between repeated measurements did not correlate with the size of δ.

**Figure 6:**
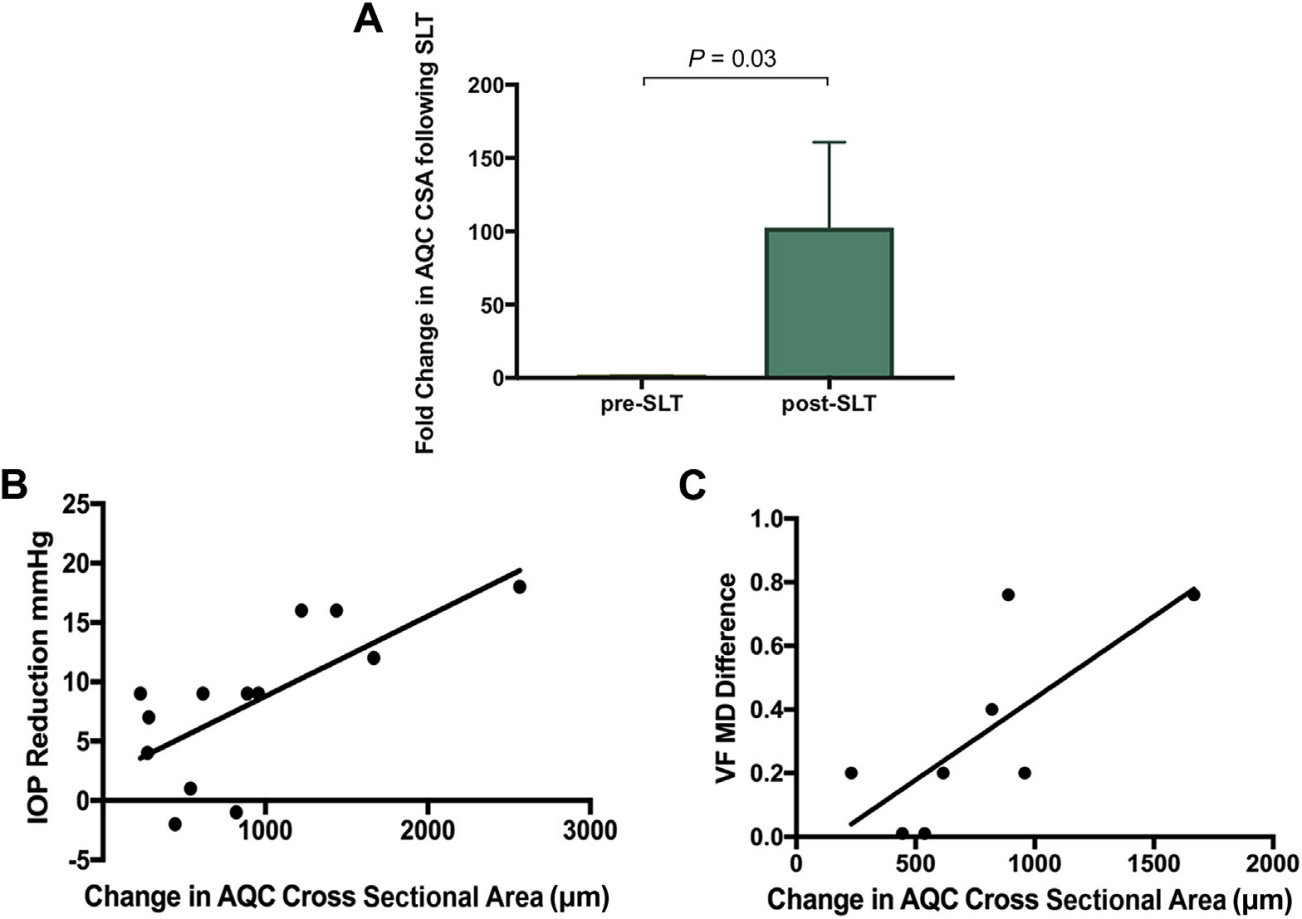
Aqueous column as a tool for quantifying aqueous outflow. **A,** Fold change in aqueous column cross-sectional area (CSA) after intervention (n = 13; *P* < 0.05; Student ratio paired *t* test). **B,** Correlation between IOP reduction and aqueous column CSA after intervention (n = 13; Pearson’s correlation coefficient 0.7; *P* = 0.007). **C,** Correlation between change in mean deviation (MD) and aqueous column CSA after intervention (n = 8; Pearson’s correlation coefficient 0.75; *P* = 0.03). AQC = aqueous column; IOP = intraocular pressure; SLT = selective laser trabeculoplasty; VF = visual field.

**Figure 7:**
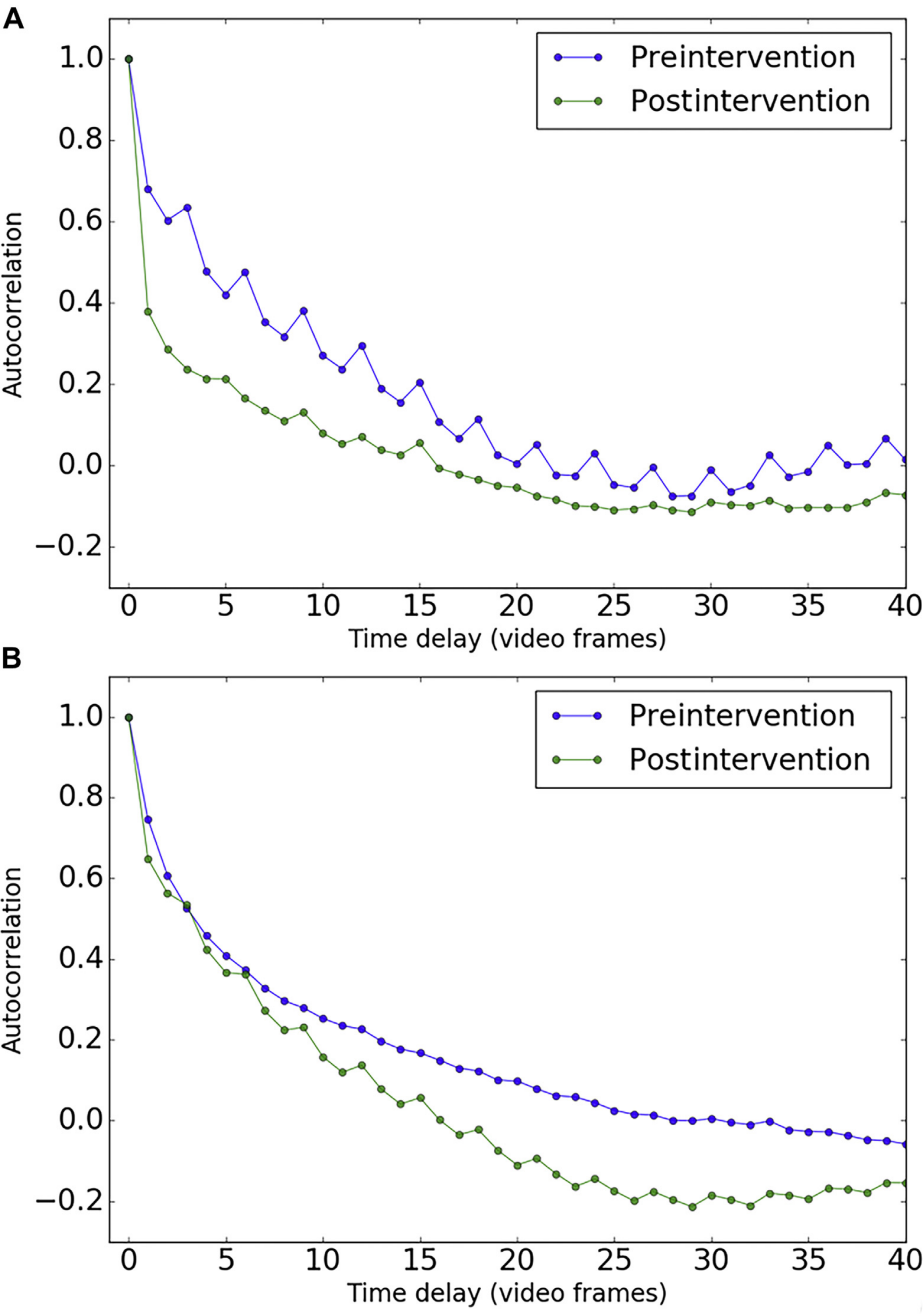
Flow rate using autocorrelation analysis before and after selective laser trabeculoplasty (SLT). **A,** Faster rate of decay is seen postintervention indicating an increase in flow rate. **B,** Similar autocorrelation decay rates seen in nonaqueous vein or background areas of the hemoglobin video imaging (HVI) images.

There was a significant increase in the aqueous column immediately after administration of our SLT protocol (Fig 6A), and this correlated with the degree of IOP reduction observed (Fig 6B) as well as with the improvement in MD observed postintervention (Fig 6C). Video 5 (available at www.ophthalmologyglaucoma.org) demonstrates individual aqueous veins from 4 patients with glaucoma before and after intervention. Figure 7 uses example 1 (Video 5) to compute an autocorrelation analysis as described earlier and using the transformation and segmentation demonstrated in Figures S1 and S2 (available at www.ophthalmologyglaucoma.org). There is an increase in the rate of decay after intervention, indicating an increase in flow rate.

## Discussion

We have used HVI to develop a method for the detailed observation and quantification of aqueous columns in episcleral venous blood. This technique can be performed noninvasively as part of a routine clinic assessment using a modified slit lamp and repeated multiple times facilitating longitudinal examination of individual patients over a period of time. We confirm previous observations on the characteristics of aqueous flow, including laminar flow, pulsatility, and altered dynamics corresponding to transient fluctuations in pressure, including the redistribution of aqueous after occlusion of an aqueous vein. We also propose a technique to compare flow rates in an aqueous vein using HVI images. The method is based on principles used in photon correlation spectroscopy and laser Doppler velocimetry where the velocity of fluids in channels is calculated by measuring fluctuations in the recorded intensity signal. When tracer particles cannot be added to maintain the physiologic parameters and monitor aqueous flow noninvasively, flow velocity estimation becomes more complex. We do not aim to provide a velocity estimate, because an accurate measurement of the decay rate would require a faster sampling rate and thus a faster frame rate camera. However, the plots derived from autocorrelation analysis may be useful as simple metrics for comparing flow in videos.

The ability to visualize and quantify physiologic aqueous flow provides us with the means to further explore the relationship between aqueous outflow and the diagnosis, monitoring, and treatment of patients with glaucoma. The correlation we have observed between well-established glaucoma parameters such as IOP and MD reinforces the use of the aqueous CSA as a tool to quantify the outflow status of an eye.

Our measurements using the SLT protocol were taken serially on the same individual within 1 hour of each other immediately before and after intervention. This enabled a direct assessment of the effects of SLT (with simultaneous administration of apraclonidine and pilocarpine) on aqueous outflow, irrespective of the known variations in outflow in a given individual during a 24-hour period. Although we have been able to quantify a change in aqueous flow after intervention, the addition of pilocarpine and apraclonidine as part of our protocol may have affected aqueous flow, and we cannot fully attribute our observed changes to SLT alone.

Establishing the variation in outflow in a healthy population is also essential before considering the use of aqueous outflow facility to assess and monitor patients with glaucoma alongside IOP and MD.

The ability to perform a dynamic assessment at high resolution while visualizing aqueous and blood in real time may help to further our understanding of the relationship between episcleral venous pressure and aqueous flow. Current tools to measure episcleral venous pressure are at best limited. Modeling the redistribution of aqueous in the presence of increased episcleral venous pressure and the turbulence at the interface between aqueous and blood using HVI may provide an estimate of the pressure in the venous circulation and should be a focus of future work in this area.

In conclusion, the HVI technique could be used more readily as described in this article during the management of those patients in whom MIGS implantation is being considered. A more precise targeting of the site of implantation, to correspond with the anatomy of an individual’s aqueous vein distribution, may improve the reliability of these devices in lowering IOP. The quantification of the aqueous column also could be used to assess the relative effectiveness of various MIGS devices as we have demonstrated for SLT. This may facilitate refinement and stratification of the choice of MIGS according to the subset of patients most likely to benefit from a particular device.

## Supplementary Data

Figure S2

Video 1 VidClip of captured images of an aqueous vein as played back before stabilization.

Video 2 VidClip of images as in video 1 after stabilization using Image J “Image Stabilizer plugin.”.

Video 3 VidClip of separate recordings of individual aqueous veins using hemoglobin video imaging (HVI).

Video 4 VidClip of pulsatile nature of aqueous flow captured using HVI. Three separate examples of displacement of aqueous after digital pressure on the globe away from the vessels of interest. Aqueous is redirected into an episcleral blood filled vessel after digital pressure on the globe with immediate resumption of usual aqueous and blood flow after release of pressure. A final example demonstrates compression of an aqueous vein using a 10/0 Vicryl loop redirects aqueous to a nearby episcleral blood vessel. Aqueous flow resumes once the maneuver is complete.

Video 5 VidClip of imaging of individual aqueous veins in 4 patients with glaucoma before and after intervention.
