# Supplementary material for: Hemoglobin Video Imaging Provides Novel In Vivo High-Resolution Imaging and Quantification of Human Aqueous Outflow in Patients with Glaucoma
Source: Ophthalmol Glaucoma. 2019 Sep-Oct;2(5):327–35. doi: 10.1016/j.ogla.2019.04.001 (PMC6876656; doi:10.1016/j.ogla.2019.04.001)
Supplement: Figure S1 [file mmc1.pdf]

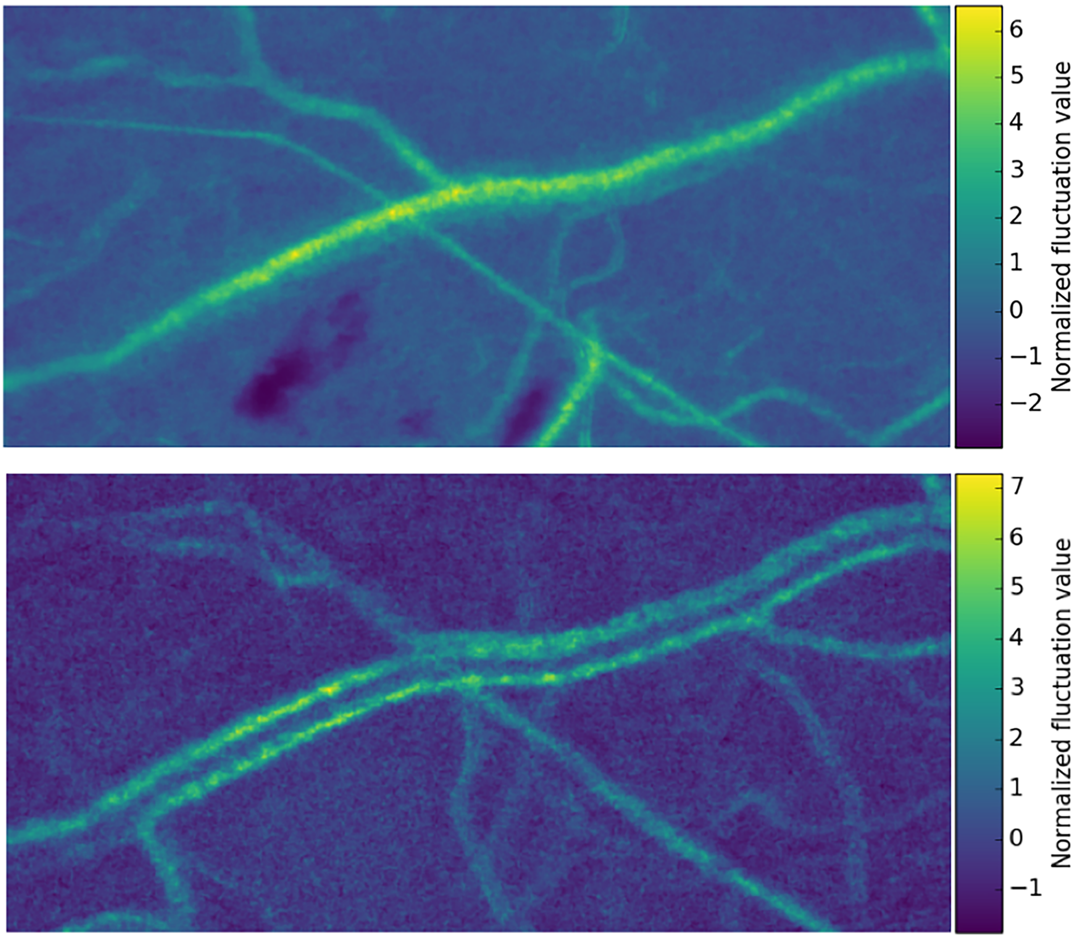

Supplementary figure 1: (Top) Pre intervention image. (Bottom) Post intervention image. Notice the relative intensity increase of the treated vessel – the contrast of the vessel relative to the background is enhanced in comparison to the original by approximately one standard deviation.
