# Supplementary material for: Hemoglobin Video Imaging Provides Novel In Vivo High-Resolution Imaging and Quantification of Human Aqueous Outflow in Patients with Glaucoma
Source: Ophthalmol Glaucoma. 2019 Sep-Oct;2(5):327–35. doi: 10.1016/j.ogla.2019.04.001 (PMC6876656; doi:10.1016/j.ogla.2019.04.001)
Supplement: Figure S2 [file mmc2.pdf]

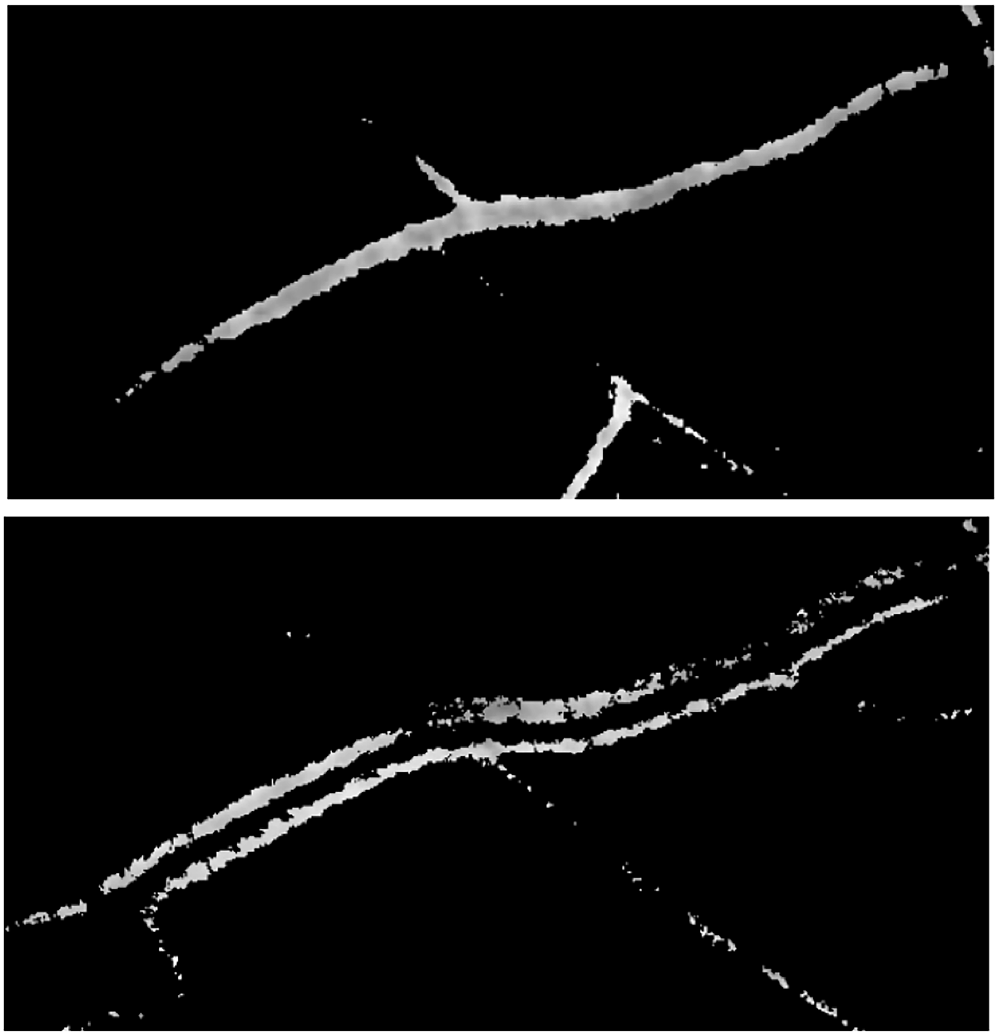

Supplementary figure 2: Example of segmented images for blood flow in an aqueous vein before intervention (top) and after intervention (bottom).
